# Relationship between Social Determinants of Health and Loneliness during COVID-19 Pandemic

**DOI:** 10.1093/geroni/igab046.2724

**Published:** 2021-12-17

**Authors:** Kalpana Padala, Christina Crawford, Clinton Gauss, Benjamin Wright, Olive Phillips, Richard Dennis, Hillary Lum, Prasad Padala

**Affiliations:** 1 Central Arkansas Veterans Healthcare System, Central Arkansas Veterans Healthcare System, North Little Rock, Arkansas, United States; 2 Central Arkansas Veterans Healthcare System, North Little Rock, Arkansas, United States; 3 University of Colorado School of Medicine, Aurora, Colorado, United States

## Abstract

Background: Older adults are most vulnerable to social isolation and loneliness during the COVID-19 pandemic compared to other populations. Risk factors for loneliness include old age, rural living, number of medical comorbidities, and poor social networks. The objectives of this study were to examine the prevalence of loneliness in older adults during COVID-19 and determine the correlation between social determinants of health and loneliness. Methods: A cross-sectional study was conducted in community dwelling older Veterans (N=132). Demographic data were collected along with variables related to social determinants of health. Loneliness data were collected with the 3-item loneliness questionnaire, and social network was assessed using the 6-item Lubben social network scale. Results: Demographic data included: mean age 73.3 (±7.5) years, 93.2% male, 53.5% rural, 84.1% Caucasian, and 13.6% African American. The majority of the participants reported loneliness (65.6%). Mean Lubben social network score was 14.6 (±6.6). There was a strong negative correlation between loneliness and social network (p<0.0001, r=-0.57; 95% CI: -0.67, -0.44). The prevalence of loneliness was significantly greater in those living alone compared to those not living alone (p=0.017; 83.9% vs. 60.6%) and those lacking internet access compared to those with internet access (p=0.025; 86.4% vs. 61.5%). Conclusion: Loneliness was found to be highly prevalent in an older cohort during the COVID pandemic. Routine inquiry about loneliness is important. Social determinants of health are likely correlated with the presence of loneliness in older adults and could be greatly impacted by policy decisions made to control community disease transmission.

